# The effects of concurrent cognitive tasks on postural sway in healthy subjects^☆^^☆☆^

**DOI:** 10.1016/j.bjorl.2015.10.011

**Published:** 2015-11-21

**Authors:** Banu Mujdeci, Didem Turkyilmaz, Suha Yagcioglu, Songul Aksoy

**Affiliations:** a Department of Audiology, Faculty of Health Sciences, Yıldırım Beyazıt University, Ankara, Turkey; b Department of Audiology, Institute of Health Science, Hacettepe University, Ankara, Turkey; c Department of Biophysics, Faculty of Medicine, Hacettepe University, Ankara, Turkey

**Keywords:** Postural balance, Task performance and analysis, Memory, short-term, Attention, Equilíbrio postural, Realização e análise de tarefas, Memória de curto prazo, Atenção

## Abstract

**Introduction:**

Keeping balance of the upright stance is a highly practiced daily task for healthy adults and is effectively performed without overt attentional control in most.

**Objective:**

The purpose of this study was to examine the influence of concurrent cognitive tasks on postural sway in healthy participants.

**Methods:**

This was a prospective study. 20 healthy volunteer subjects were included. The cognitive and balance tasks were performed separately and then, concurrently. Postural control task consisted of 6 conditions (C) of the Sensory Organization Test. The cognitive task consisted of digit rehearsal task of varying presentation and varying levels of difficulty.

**Results:**

A statistically significant difference was noted between dual task and no task for C1, C2, C3 and C4 Sensory Organization Test scores (*p* < 0.05). There was no statistically significant difference between dual task versus non-task for C5, C6 and combined Sensory Organization Test scores (*p* > 0.05).

**Conclusion:**

During dual task, increase has been determined in postural sway for C1, C2, C3 and C4 for all presentation modes and difficulty levels of the cognitive tasks.

## Resumo

**Introdução:**

Manter o equilíbrio na postura vertical é uma tarefa cotidiana constantemente praticada por adultos saudáveis, e é efetivamente realizada pela maioria sem a necessidade de um controle atencional.

**Objetivo:**

A finalidade deste estudo foi examinar a influência de tarefas cognitivas concomitantes no equilíbrio postural de indivíduos saudáveis.

**Método:**

Trata-se de um estudo prospectivo em que participaram 20 voluntários saudáveis. As tarefas cognitivas e de equilíbrio foram realizadas separadamente; e em seguida, realizadas simultaneamente. A tarefa de controle postural consistiu em seis condições (C) do Teste de Organização Sensorial (TOS). A tarefa cognitiva consistiu na repetição de dígitos com apresentações variadas e vários níveis de dificuldade.

**Resultados:**

Houve diferença estatisticamente significante entre os escores do TOS para as condições C1, C2, C3 e C4 para “dupla tarefa” *vs.* “não tarefa” (p < 0,05). Não houve diferença estatisticamente significante entre os escores do TOS para as condições C5 e C6 e para a combinação dos escores do TOS para “dupla tarefa” *vs.* “não tarefa” (p > 0,05).

**Conclusão:**

Durante a realização de dupla tarefa, foram determinados aumentos na oscilação postural para as condições C1, C2, C3 e C4 para todos os modos de apresentação e níveis de dificuldade das tarefas cognitivas.

## Introduction

Stance balance control is a complex motor skill that relies on the interactions among three major sensory components: the visual, somatosensory, and vestibular systems.^1^ The integration of visual, vestibular, and somatosensory components is used to maintain one's postural balance. Postural control represents a complex interplay between the sensory systems which involves perceiving environmental stimuli, responding to alterations, and maintaining the body's center of gravity within the base of support.^2^ Keeping balance of the upright stance is a highly practiced daily task for healthy adults and is effectively performed without overt attentional control in most circumstances.^3^

The nature of the relation between postural control and cognition remains unclear.^4^ The main questions are whether the postural challenge will have an effect on cognitive performance, and conversely, whether performance of the cognitive task decreases postural stability.^5^ Several investigators have suggested that control of stance and control of locomotion require some level of higher cognitive processing despite their highly practiced nature.^6^, ^7^, ^8^ The methodology for experimental testing using a dual-task paradigm with motor skills is described in a classic paper by Abernethy.^9^ One of two tasks is designated the primary task. Primary task performance is maintained at the baseline level during the dual task condition.^10^ The dual task paradigm provides information on the automaticity, hemispheric locus and structural independence of processes hypothesized to underlie the production of skilled performance.^9^

Common real-word observations of people conversing while walking or listening to music while running illustrate this statement. In those situations, the attentional resources must be divided to properly perform both tasks.^11^ If, in the dual task condition, performance on the secondary task is reduced from the baseline level, it reflects high attentional demands of the primary task and suggests insufficient reserve capacity to perform the secondary task at the baseline level.^10^ A commonly accepted approach to understanding dual-task interference between motor and cognitive tasks is grounded in limited resource or capacity theories of attention. According to those theories, the brain's information-processing capacity or resources available for processing are limited.^12^ A multiple-resource theory would predict dual-task interference only when concurrent tasks employ aspects of the same cognitive resource.^13^ The combined difficulty of the tasks requires excessive attention, then interference between tasks could occur. That is, the quality of performance of both tasks could decrease, or one task be performed in preference to the other.^8^, ^14^

The purpose of this study was to examine the influence of different concurrent cognitive tasks on postural sway in healthy subjects. We hypothesized that postural sway would be modulated by the presence and difficulty of digit rehearsal task.

## Methods

### Participants

Twenty subjects (10 women, 10 men; mean age = 22.40 ± 4.46) were recruited to participate in this study. Inclusion criteria required for subjects: no history of neurological disease, hearing impairments, visual impairments not correctable with lenses, musculoskeletal impairments and injuries or disorders affecting balance.

### Tests

All measurements were carried out in one experimental session for each subject. At first, the cognitive and balance tasks were performed separately (single task); then the cognitive and balance tasks were performed concurrently (dual task). The study was approved by the Ethical Committee of the institution. After the scope and objective of the research had been explained to the subjects who participated in the research, their written consents were also obtained.

### Balance test

All subjects were evaluated in a study of postural stability using Computerized Dynamic Posturography. The Sensory Organization Test (SOT) was conducted with a NeuroCom Smart Balance Master (NeuroCom International, Inc., Clackamas, OR). The SOT was performed in a clinically routine manner. Six test conditions were developed for balance assessment: eyes open fixed support surface and surround (fixed–fixed) (C1), eyes closed fixed support surface and surround (fixed–absent) (C2), eyes open fixed support surface and sway-referenced surround (fixed–sway) (C3), eyes open sway-referenced support surface and fixed surround (sway–fixed) (C4), eyes closed sway referenced support surface and fixed surround (sway–absent) (C5), eyes open sway referenced support surface and surround (sway–sway) (C6). Sway gain was set at 1.0, exactly matching sway referencing to subject's sway as described in the NeuroCom System Operator's Manual.^15^ The length of each trial was extended to 20 s. The subjects then completed each of the six conditions three times.

### Cognitive test

Before beginning the balance task, participants completed the digit memory test so that we could determine each participant's digit string. The digits were given using E-Prime 2.0 Professional (Psychology Software Tools Inc., Pittsburgh, PA, USA). The digits were given at the rate of one per second on a computer of NeuroCom Balance Master via E-Prime 2.0 Professional using 144-point Times font positioned at eye level in front of the participant. The participants were given a test of digit string using auditory, visual and auditory–visual mixed presentation, and they were asked to repeat the order. Speakers were used for auditory presentation. When a subject failed at both attempts at one level, the test ceased. The maximum number of digits correctly recalled in the same order as presented served as the number of digits displayed in the difficult experimental condition of the cognitive task. We determined the number of digits displayed in the easy condition by taking half of the maximum number of digits recalled. After a relaxation break of ten minutes, dual task order was applied to the subjects.

### Procedures

**Figure 1:**
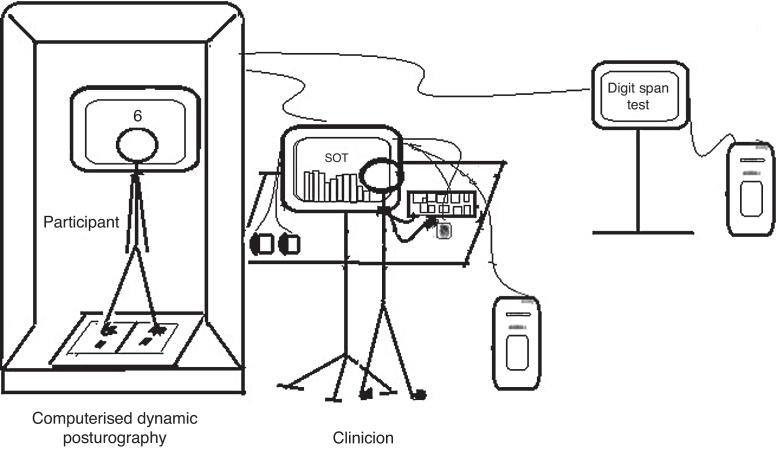
Dual task equipment.

The dual task session began with instructions. After confirming that the participant understood the procedure, testing began. The participant was then instructed to stand on the NeuroCom Balance Master platform and to perform the cognitive test while maintaining balance under six test conditions (Fig. 1). Each trial was randomized to minimize practice effects.

**Figure 2:**
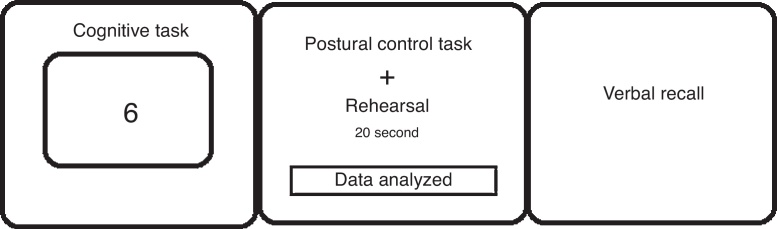
Illustration of the dual task procedure.

The participants were given a test of digit string using auditory, visual and auditory–visual mixed presentation. The order of presentation of trials in each of the experimental conditions (no-task, easy, difficult) was fully randomized. Three trials in each of the six SOT conditions were presented in random order. On each trial in the digit-task conditions, digit string was displayed on a computer. The digits were given at the rate of one per second. Participants were instructed to rehearse the digit string until it disappeared and a blue screen appeared. Meanwhile participants had to encode this digit string until disappeared. At that point, the 20 s postural sway measurement period began. For the eyes-closed trials, participants were instructed to close their eyes; for eyes-open position participants kept their eyes open, and were instructed to simply look ahead but were not told to fixate on any given object or location. Subjects had to mentally repeat the string during the 20 s duration. At the end of this 20 s maintenance period, participants repeated digit string (Fig. 2). Participants’ responses were recorded for each trial. The number of errors (intrusion, order error and omission) in the response was recorded by the experimenter. All of the scores recorded within the 20 s SOT test were used in the statistical analyses. The subjects were allowed to take a relaxation break of ten minutes after each dual task stage, which sums up to a total time of fifty minutes. During the relaxation breaks, the subjects were offered refreshments. Although it changed depending on the cognitive performance of the subject, the duration of all tests, including the relaxation breaks, ranged between 130 and 150 min.

### Data analysis

Statistical analyses were performed using the SPSS software version 18. The Wilcoxon test was used to assess differences between mean balance scores obtained under the no-task and dual-task conditions (easy, difficult) for each of the six balance conditions and each presentation (auditory, visual, auditory–visual mixed). Descriptive analyses were presented using medians and interquartile range (IQR) for the non-normally distributed and ordinal variables. A *p*-value of less than 0.05 was considered to show a statistically significant result.

## Results

The SOT scores obtained from the dual task conditions were compared separately with the no-task SOT scores. There was statistically significant difference between dual task and no-task C1, C2, C3 and C4 SOT scores (*p* < 0.05). There was no statistically significant difference between dual task and no-task SOT scores for C5, C6 and combined SOT scores (*p* > 0.05) (Table 1).

**Table 1:** The comparison of the no-task SOT scores and SOT scores obtained simultaneously with cognitive task.

Task	SOT			Scores			
	C1	C2	C3	C4	C5	C6	Composite
*No task*							
Median	94.50	93.00	92.33	87.50	68.66	70.83	81.00
IQR	1.83	3.17	5.17	6.00	12.75	17.25	7.00

*Auditory easy*							
Median	89.33	89.16	90.00	80.83	72.83	68.83	80.50
IQR	7.25	4.50	6.58	12.42	12.67	21.33	6.50
*p*	0.001^a^	0.001^a^	0.005^a^	0.002^a^	0.355	0.601	0.256

*No task*							
Median	94.50	93.00	92.33	87.50	68.66	70.83	81.00
IQR	1.83	3.17	5.17	6.00	12.75	17.25	7.00

*Auditory difficult*							
Median	92.16	91.00	89.83	81.66	71.00	71.66	82.00
IQR	6.25	6.25	7.92	11.75	11.00	20.92	8.00
*p*	0.001^a^	0.003^a^	0.016^a^	0.003^a^	0.588	0.679	0.615

*No task*							
Median	94.50	93.00	92.33	87.50	68.66	70.83	81.00
IQR	1.83	3.17	5.17	6.00	12.75	17.25	7.00

*Visual easy*							
Median	90.50	89.00	88.16	81.83	71.00	71.50	79.50
IQR	9.00	9.25	5.50	10.00	13.75	24.08	11.75
*p*	0.000^a^	0.006^a^	0.002^a^	0.007^a^	0.904	0.478	0.055

*No task*							
Median	94.50	93.00	92.33	87.50	68.66	70.83	81.00
IQR	1.83	3.17	5.17	6.00	12.75	17.25	7.00

*Visual difficult*							
Median	90.33	90.33	90.16	79.33	75.66	68.00	78.50
IQR	6.17	8.08	7.67	9.83	16.00	17.75	8.50
*p*	0.000^a^	0.001^a^	0.017^a^	0.001^a^	0.287	0.856	0.126

*No task*							
Median	94.50	93.00	92.33	87.50	68.66	70.83	81.00
IQR	1.83	3.17	5.17	6.00	12.75	17.25	7.00

**Auditory–visual**							
*Mixed easy*							
Median	90.66	90.50	89.83	82.50	73.33	63.00	79.50
IQR	6.42	7.17	8.42	13.67	16.42	19.92	8.75
*p*	0.001^a^	0.001^a^	0.007^a^	0.015^a^	0.370	0.185	0.055

*No task*							
Median	94.50	93.00	92.33	87.50	68.66	70.83	81.00
IQR	1.83	3.17	5.17	6.00	12.75	17.25	7.00

**Auditory–visual**							
*Mixed difficult*							
Median	92.16	89.83	89.33	80.00	72.00	66.66	80.00
IQR	7.25	5.42	8.83	20.08	15.17	13.92	7.25
*p*	0.001^a^	0.000^a^	0.006^a^	0.013^a^	0.411	0.411	0.204

SOT, Sensory Organization Test; C, condition; IQR, interquartile range.

a Statistically significant at *p* < 0.05.

Within the conditions of dual task of the same difficulty degree, as a result of double comparison according to the presentation manner of the stimulus (e.g. auditory easy and visual easy) no statistically significant difference was obtained within all dual task condition SOT scores (*p* > 0.05).

In the dual task order, as a result of double comparison according to the difficulty level of the cognitive tasks having the same presentation manner (e.g. auditory easy and auditory difficult) no statistically significant difference was obtained within all dual task condition SOT scores (*p* > .05).

No statistical analyses were carried out for the mistakes of the subjects in the six SOT conditions that were done with three repetitive in the dual task order; only their average was calculated. Six errors were made in the auditory easy condition, six in the auditory difficult condition, 12 in the visual easy condition, 98 in the visual difficult condition, five in the auditory–visual mixed easy condition, and 117 errors were made in the auditory–visual mixed difficult condition.

## Discussion

The purpose of this study was to examine the influence of concurrent cognitive tasks on postural sway in healthy participants. Our short-term memory task focused on the process of rehearsal. In dual task studies, visual and/or auditory cognitive tasks generally have been used simultaneously with postural task,^16^, ^17^, ^18^ but there are no studies using auditory–visual mixed cognitive task. Therefore, in this study, auditory–visual mixed task, in addition to visual and/or auditory cognitive tasks with concurrent postural task has been also used during dual task. No-task scores and dual task scores were obtained separately and compared.

In this study, anterior–posterior sway increased for C1, C2, C3, C4 SOT scores but was not affected for C5, C6 and combined SOT scores compared to no-task scores. During dual task, together with adding the cognitive tasks to postural control task, statistically significant increase has been determined in the postural sway for relatively easy postural conditions C1, C2 (the platform is stable), C3 and C4 (only one of the proprioceptive and visual perception input distorted or absent) for all of the presentation manners and difficulty levels of the cognitive tasks. This finding is consistent with the results of Pellecchia et al.^19^ reporting that postural sway increased when dual task was applied. Other investigators^8^, ^20^, ^21^, ^22^ have also found deficits in postural control following the addition of a cognitive task. Several reasons can be offered for this finding. Increased postural sway under dual task in this study can be explained by divided attention.^23^ It is known that when one needs to maintain the balance of upright stance while performing a concurrent cognitive task, attention is divided between postural and cognitive tasks.^3^ During a dual task, postural and cognitive tasks can also be in competition for central processing or attentional resources, causing a reduction in performance of either task.^23^, ^24^ This may also account for increase in postural sway in this study.

The possibility that participants might have employed a strategy that maintained cognitive performance at the expense of postural control during dual task^25^ could relate a possible explanation for postural sway increase in this study. As another reason for higher postural sway during dual task condition in relatively easy postural task conditions (C1, C2, C3, C4) compared to single task performance, it can be thought that participants may have focused singularly on postural task or have ordinarily directed some amount of attention to postural control, but it has a detrimental effect. It can be concluded that focusing intentionally on postural control during dual task resulted in a less automatic control of balance, thus disturbing the efficiency of postural control. Hunter and Hoffman^16^ have suggested that focusing solely on a balance task could possibly lead to an increase in muscle tension, leading to increased joint stiffness and rigidity in the body, that in turn may result in increased postural sway. Likewise, according to constrained action,^26^ focusing attention on a highly automatized behavior such as postural control interferes, rather than helps, the automatic control process.^3^

When healthy humans are perturbed, while standing on a movable platform, a cascade of balance-correcting muscle reactions occurs. Some balance corrections have been termed “automatic”, whereas others, which approximate voluntary reactions. Balance corrections can be provided by knee movements as a secondary contribution or as part of the correcting strategy itself.^27^ Knee proprioception is presumed to be required for protection against excessive movements, stabilization during static posture and coordination of movements.^28^ We did not evaluate whether proprioceptive feedback from the knees contribute or not to the triggering of balance corrections. Therefore, it is not possible to make an explanation about knee movements on postural control. In a study, Oude Nijhuis et al.^29^ have examined the effect of bilateral knee flexion on automatic balance corrections generated by sudden perturbations. They have found that healthy adults can incorporate voluntary knee flexion into their automatic balance corrections and that this depends on the direction of the postural perturbations.^29^ Further studies evaluating knee flexions on postural balance during secondary task can be made.

The finding showing increase in postural sway during dual task for four of the six SOT conditions in this study was in opposition with the previous researches using SOT evaluation.^30^, ^31^, ^32^ Broglio et al.^30^ reported significant improvements in SOT conditions 1, 3, 4. Resch et al.^31^ found decrease in postural sway for SOT conditions 1 and 2, and Teel et al.^32^ explained sway decrease for SOT condition 1. Other investigators^3^, ^16^, ^17^, ^18^, ^24^, ^33^ who did not use SOT have also reported increased postural stability under dual task conditions. The discrepancies between our study and the other studies that have or have not used SOT conditions may be explained by cognitive task differences used in these studies.^24^ The different cognitive tasks may have challenged the brain's ability to divide attention differently. It is known that some cognitive tasks may allow more allocation to postural control mechanism, resulting in an increase in equilibrium balance score.^32^

Our findings obtained from this study partially support the study hypothesis (for C1, C2, C3, C4), assuming that addition of cognitive task to postural task will have an effect on postural stability. However, they do not support the hypothesis that difficulty of cognitive task will have an effect on postural stability under dual task.

In the comparison of the no-task SOT scores with dual task SOT scores C5, C6 in which both of the visual and proprioceptive perception inputs are absent or distorted, anterior–posterior sway has not been affected (*p* > 0.05). C5 and C6 are the most difficult postural conditions. The same result was found for combined SOT score. This result is similar to that obtained by Barin et al.,^34^ who did not find a significant difference in postural sway in young adults when performing subtraction tasks under altered sensory conditions. Similarly, in dual task studies using SOT conditions postural stability did not change with sensory conditions.^30^, ^31^, ^32^, ^35^ Postural sway was essentially unaffected by concurrent cognitive activity for SOT scores C2, C4, C5, C6,^31^ SOT scores C1, C3, C6,^32^ SOT scores C6,^30^ and SOT scores C1 to C6.^35^

One explanation for unaffected postural sway even in the most difficult postural conditions with the addition of secondary cognitive task in this study may be that participants have switched attention from internal focus (balance) to external focus (cognitive).^3^, ^10^ Releasing postural control from attentional focus when attention directed toward a concurrent task might have allowed postural control to work in a more automatic, efficient manner.^16^, ^18^ Wulf et al.^26^ have reported that an external focus of attention promotes the use of more automatic control processes in the brain, as opposed to voluntary muscle control as with the internal focus to correct postural perturbation.

Shumway-Cook and Woollacott^35^ have explained similar postural stability between single task and dual task, as attentional demands of maintaining balance stability are fairly constant across sensory conditions. This explanation also can be valid for unchanged postural sway even in the most difficult sensory SOT conditions 5 and 6 in our study.

Another possible explanation for unaffected postural sway under dual task may be related to the conditions themselves.^32^ Under SOT conditions 5 and 6, which are, the participant may have already been dividing his/her attention between balance and surrounding during the single task paradigm due to the moving platform and surroundings as reported by Teel et al.^32^

A physiological explanation of our findings is that cerebral processing during dual-task conditions apparently modifies how the central nervous system controls postural stability. Under normal conditions, balance is controlled via integration of sensory information provided by the visual, vestibular, and somatosensory systems.^30^ Input based on limb positioning is transmitted to the basal ganglia. This signal is integrated with planned actions developed in the premotor cortex and supplementary motor cortex in the cerebellum. The descending pathway continues via alpha motor neurons, which innervate skeletal muscle, allowing for regulation of balance.^30^, ^36^ Typically, the visual and somatosensory inputs provide the majority of information to maintain postural stability.^30^, ^37^

Postural sway during dual task conditions can be detectable using different test protocols like Bertec force platform,^17^ Kistler force platform^16^ or AMTI Accusway System for Balance and Postural Sway Measurement.^19^ These tests quantify sway range and variability in both anterior–posterior direction and medial–lateral (side to side) direction. According to SOT protocol used in the present study, postural sway could only been detected in anterior–posterior direction like the other studies using SOT.^21^, ^30^, ^35^ Therefore, in this study, the effect of concurrent cognitive tasks on postural sway in medial–lateral direction is not known and comparisons with the results of the other studies could not be made. In the study of Pellechia et al.^19^ postural sway in medial–lateral direction has not been affected from secondary cognitive tasks; anterior–posterior sway has increased when the difficulty of concurrent cognitive task increased. Riley et al.^17^ have found decreased medial–lateral postural sway variability as the cognitive load imposed by a short term memory task increased. Hunter and Hofmann^16^ have explained reduced level of medial–lateral center of pressure motion with a secondary cognitive task.

In this study, no statistically significant difference was observed between the presentation manners or difficulty levels of simultaneous cognitive tasks and postural sway (*p* > 0.05). This result may suggest that the type of stimuli and difficulty of cognitive task are rather unspecific and independent of postural control.^18^ This result is different from those of Riley et al.,^17^ explaining that auditory task affects postural sway more than visual task. Contrary to our finding, Pellecchia et al.^19^ reported the more impacted postural sway with the more difficult cognitive task. On the other hand, in accordance with our result, it has been reported that there was no effect of the difficulty of cognitive task^18^ and similar effects of type of stimuli^16^, ^17^, ^18^ on postural sway.

In our study, when cognitive task paired with postural task simultaneously, it was determined that the most mistakes were made with visual difficult and auditory–visual mixed difficult dual task conditions, while the least mistakes were made with auditory cognitive task. This finding with respect to visual and auditory task is consistent with the study of Penney,^38^ which reported a large auditory modality advantage in serial recall that suggests a certain greater robustness or persistence of acoustic memory compared to visual memory. However, Riley et al.^17^ found more cognitive performance errors during auditory task than visual task. The reason for the visual and auditory cognitive task performance errors seen in this study is thought to be explained by the modality effect. The term modality effect referred to the finding that, in short-term memory tasks, auditory presentation almost always resulted in higher recall than did visual presentation.^38^ Since information about the effect of auditory–visual mixed task concurrent postural task on postural sway is not available in the literature, no comparison can be made and a possible explanation for auditory–visual mixed task errors seen in our study cannot be made. Further studies are necessary to detect the reason of increase in cognitive errors with auditory–visual mixed task concurrent postural task on postural sway.

The limitations of our study are: the limited number of subjects that participate in the study, the fact that only the rehearsal phase is evaluated and not being able to assess the encoding phase and response time for cognitive task.

## Conclusion

As a result, within the dual task order, together with adding the cognitive tasks to postural control task, a statistically significant increase has been determined in the postural sway for C1, C2, C3 and C4, but no significant difference has been observed within the sway for C5 and C6, which are the most difficult postural tasks. Under dual task, it is determined that the presentation manner and difficulty level of the stimulus within the cognitive tasks have no effect on postural sway. When cognitive task paired with postural task simultaneously, the most mistakes were found with visual difficult and auditory–visual mixed difficult dual task conditions, while the least mistakes were made with auditory cognitive task. Vestibular impairments increase the requirement of attention for postural control.^39^ In addition, attentional capacity in multi-tasking conditions decreases with aging.^40^ Older adults may be at risk for falls under concurrent task conditions.^7^ It is thought that the dual task model used in this study may provide useful information about the assessment of the dual task capacity among geriatric subjects and with subjects with vestibular disorders.

## Conflicts of interest

The authors declare no conflicts of interest.
